# Evidence for a Role of srGAP3 in the Positioning of Commissural Axons within the Ventrolateral Funiculus of the Mouse Spinal Cord

**DOI:** 10.1371/journal.pone.0019887

**Published:** 2011-05-31

**Authors:** Claire Bacon, Volker Endris, Irwin Andermatt, Vera Niederkofler, Robert Waltereit, Dusan Bartsch, Esther T. Stoeckli, Gudrun Rappold

**Affiliations:** 1 Department of Human Molecular Genetics, University of Heidelberg, Heidelberg, Germany; 2 Institute of Molecular Life Sciences, University of Zürich, Zürich, Switzerland; 3 Department of Molecular Biology, Central Institute of Mental Health, University of Heidelberg, Mannheim, Germany; Institut de la Vision, France

## Abstract

Slit-Robo signaling guides commissural axons away from the floor-plate of the spinal cord and into the longitudinal axis after crossing the midline. In this study we have evaluated the role of the Slit-Robo GTPase activating protein 3 (srGAP3) in commissural axon guidance using a knockout (KO) mouse model. Co-immunoprecipitation experiments confirmed that srGAP3 interacts with the Slit receptors Robo1 and Robo2 and immunohistochemistry studies showed that srGAP3 co-localises with Robo1 in the ventral and lateral funiculus and with Robo2 in the lateral funiculus. Stalling axons have been reported in the floor-plate of *Slit* and *Robo* mutant spinal cords but our axon tracing experiments revealed no dorsal commissural axon stalling in the floor plate of the *srGAP3* KO mouse. Interestingly we observed a significant thickening of the ventral funiculus and a thinning of the lateral funiculus in the *srGAP3* KO spinal cord, which has also recently been reported in the *Robo2* KO. However, axons in the enlarged ventral funiculus of the *srGAP3* KO are Robo1 positive but do not express Robo2, indicating that the thickening of the ventral funiculus in the *srGAP3* KO is not a Robo2 mediated effect. We suggest a role for srGAP3 in the lateral positioning of post crossing axons within the ventrolateral funiculus.

## Introduction

Commissural axons in the developing mouse spinal cord are drawn ventrally to the midline by attractive guidance cues secreted by floor plate cells. Upon reaching the floor plate, commissural axons switch their responsiveness from attractive to repulsive guidance cues in order to cross and leave the midline, projecting longitudinally at the contralateral side [1]. Axons projecting longitudinally in more medial positions (i.e. closer to the floor plate) form the ventral funiculus and axons projecting in the longitudinal plane at more lateral positions (further away from the floor plate) form the lateral funiculus. Numerous repulsive guidance factors have been identified that repel commissural axons away from the midline into their correct ventrolateral positions including the ligand Slit and its Robo receptors [1], [2], [3], [4].

Complete loss of all three *Slit* genes leads to the failure of commissural axons to properly leave the midline and extend into the lateral funiculus [2]. The Slit receptors Robo1 and Robo2 also have important functions in commissural axon guidance. In *Robo1* KO and *Robo1/Robo2* double KO mice, commissural axons stall within the floor-plate [2], [4] resulting in a significant reduction of the ventral funiculus area [4]. Commissural axons in *Robo2* KO mice do not stall within the floor-plate but project longitudinally in closer proximity to the floor-plate on the contralateral side, resulting in a thickening of the ventral funiculus [4] and a thinning of the lateral funiculus [2], [4]. It is now accepted that Robo1 prevents axon stalling during crossing and guides the longitudinal projection of post-crossing axons at ventral positions whereas Robo2 guides the longitudinal projection of commissural axons in more lateral positions [2], [4].

Repulsion of axons from the midline depends upon dynamic reorganisation of the growth cone cytoskeleton. The Rho GTPases are key regulators of the actin cytoskeleton and have been implicated in axon guidance downstream of Slit Robo signaling [5]. The active (GTP-bound) and inactive (GDP-bound) state of RhoGTPases are controlled by guanine nucleotide exchange factors (GEFs) and GTPase activating proteins (GAPs). GEFs and GAPs act antagonistically with each other; GEFs catalyse nucleotide exchange for activation, while GAPs promote GTP hydrolysis, leading to inactivation.

In a yeast two-hybrid screen, the Slit Robo GTPase activating proteins (srGAPs) were identified as a family of GAPs that interact with the intracellular domain of Robo1 [6]. The srGAP family has three members – srGAP1, srGAP2 and srGAP3. SrGAP proteins have previously been implicated in synaptic plasticity and neuronal migration [6], [7], [8]. However, the role of srGAP proteins in axon guidance remains to be examined.

We investigated dorsolateral commissural axon crossing in a *srGAP3* KO mouse. No axon stalling was observed within the floor-plate of *srGAP3* KO mice. Using L1 staining we revealed that the ventral funiculus of *srGAP3* KO spinal cords is significantly thicker compared to wild type (WT), while the lateral funiculus is significantly thinner. Our results point to a role for srGAP3 in the lateral positioning of post-crossing commissural axons within the ventrolateral funiculus.

## Materials and Methods

### Ethics statement

Pregnant mice were sacrificed by cervical dislocation prior to embryo removal, in strict accordance with a protocol approved by the Animal Protection Committee at the University of Heidelberg.

### Animals

A detailed description of the generation and phenotype of the *srGAP3* KO mouse will be described elsewhere. In brief, to mimic the break of the *srGAP3* gene described by Endris at al. [9], two consecutive stop codons were introduced at the end of exon 3 of the murine *srGAP3* gene by homologous recombination in ES cells. A targeting construct containing 5 kb upstream and 2 kb downstream of exon 3 was derived from the PAC RPCIP711J22374Q2 (library RPCI-21, RZPD, Germany) and was enhanced by TK-cassette for negative selection on the 3′end and frt flanked kanamycin resistance cassette (neo) immediately following exon 3. Targeted ES-cells were injected into blastocysts of C57BL/6J mice and implanted into pseudopregnant recipients. Chimaeras were crossed with C57BL/6J mice and the offspring was analyzed for germ line transmission by PCR. To remove the frt-flanked neo-cassette, *srGAP3*-stop knock-in mice were crossed with Tg(ACTFLPe)9205Dym/J (S.Dymecki, USA). Founder *srGAP3*-stop knock-in mice were backcrossed with C57BL/6J mice for six to eight generations. Loss of srGAP3 protein in brain tissue was confirmed by western blot.

### Co-immunoprecipitation

Immunoprecipitations were carried out using standard procedures. Briefly, HEK293 cells (Invitrogen) transiently transfected with full length srGAP3 and Myc-tagged Robo1/2 constructs were lysed in modified RIPA buffer (50 mM Tris/HCl pH 7.4, 1% NP-40, 0.25% Na-deoxycholate, 1 mM EDTA, 2 mM Na_3_VO_4_, 2 mM NaF) supplemented with protease inhibitor mix G (Serva). Cell lysates (400 µg) were incubated with a rabbit srGAP3 (19.1) antibody (Pineda Antibody Service, Berlin, Germany) or an unrelated control antibody. Immunocomplexes were precipitated using Ultralink protein A/G sepharose (Pierce) and separated by SDS-Page together with 20 µg of total cell extracts as a control, before blotting onto PVDF membranes (Immobilon-FL, Millipore). Western blots were blocked with 50% Odyssey Blocking Buffer in TBS and incubated with primary antibody solutions (anti-myc 9E10, Calbiochem) in blocking buffer including 0.1% Tween. Membranes were washed in TBS-T followed by secondary antibody incubation (fluorophore-labelled IRDye 680 and 800CW) in blocking buffer. Blots were scanned with a LI-COR imager. srGAP3 SH3 mutants were generated as previously described [10].

### Immunohistochemistry and quantification of commissural axon tracts

Embryos were fixed and cryosectioned as previously described [11]. Slides of transverse E12.5 cryosections were rinsed briefly in PBS and then blocked for 1 hour at RT in 5% goat serum/0.1% triton in PBS (for TAG-1 staining) or 5% horse serum/0.1% triton in PBS. Slides were then incubated in primary antibodies, diluted in block solution for 2 hours at RT at the following dilutions: rabbit anti-srGAP3 (Sigma Aldrich, Steinheim, Germany) 1∶2000, goat anti-Robo1/goat anti-Robo2 (R&D Systems) 1∶500, rat anti-L1 (MAB5272, Chemicon) 1∶2000. For TAG-1 staining, slides were incubated overnight in 4D7 supernatant (Developmental Studies Hybridoma Bank, University of Iowa, USA). Slides were then washed in PBS and incubated in Alexa-fluorophore-conjugated secondary antibodies (Molecular Probes, Oregon, USA) at 1∶800 dilution in block solution for 1 hour at RT in the dark. For TAG-1 staining, a goat anti-mouse IgM was used at 1∶300 dilution for two hours. For Robo1 and Robo2 staining, slides were incubated in a horse anti-goat biotinylated secondary antibody (Vector Laboratories) at 1∶500 dilution in block solution for 1 hour at RT. After secondary antibody incubation, Robo1 and Robo2 staining was enhanced using a tyramine signal amplification system (Perkin Elmer, Boston, USA) according to manufacturers instructions. All slides were mounted in Dako fluorescent mounting medium and imaged on a Nikon C1-CLEM confocal microscope or a Nikon 90i microscope, using a Nikon DS IQM black and white camera. Brightness and contrast of images was optimised using ImageJ software.

For measuring the area of the ventral funiculus, transverse cryosections were stained with L1 to allow visualisation of the axon tracts. The region for quantification was 100 µm from the edge of the floor plate and this defined region was traced and the area quantified using ImageJ software. To exclude the possibility that any phenotype observed in srGAP3 KO embryos is due to a developmental delay, we always compared WT and srGAP3 KO littermates. Additionally, all measurements were normalised to the total area of the spinal cord. For quantification of the lateral funiculus, the entire L1-positive ventrolateral area was quantified and the ventral funiculus area was subtracted to get the lateral funiculus area, which was normalised to the whole spinal cord area. Quantifications of cervical, thoracic and caudal sections were performed separately as commissural axon crossing begins in the cervical spinal cord and progresses caudally. L1 staining intensity per area was measured using the ImageJ histogram tool and did not differ in the ventrolateral funiculus suggesting that there was no marked difference in the number or density of fibers. All images were captured using the same exposure times and brightness and contrast of images was not altered before quantifications of the intensity were made.

### DiI tracing of commissural axons

E12.5 mice embryos were eviscerated and pinned onto Sylgard plates before fixing in 4% paraformaldehyde for 1 hour at RT. After fixation, spinal cords were removed and prepared in an open book configuration. Dorsolateral commissural axons were traced by the injection of the carbocyanide dye DiI (5 mg/ml; Molecular Probes) into the area of the cell bodies in the dorsal spinal cord. Injected open books were left in the dark for 48 hours to allow the DiI to anterogradely label the commissural axon tracts before mounting in PBS. Commissural axons were visualised by fluorescence microscopy and each injection site was classified as being normal or containing more than 50% of the axons stalling in the floor plate, at the exit site or both.

### RNA probe synthesis and *in situ* hybridisation

srGAP1, srGAP2 and srGAP3 probe synthesis was performed as previously described [11]. For Robo1 probe synthesis, mouse cDNA was used as a template for PCR. A 660 bp PCR product was generated using forward primer 5′–TCCCCACC TCATACTTACGG-3′ and reverse primer 5′-GATCATCTGCGTAGGCTTCC-3′. The resulting PCR product was cloned into the pST-blue Acceptor Vector (Novagen). *In vitro* transcription of the Robo1 probe was carried out using Megascript T7 (antisense) and SP6 (sense) High Yield Transcription Kit (Roche Diagnostics, Germany). *In situ* hybridisation for *srGAP1, srGAP2, srGAP3* and *Robo1* on transverse cryosections of embryonic mouse spinal cord was performed as previously described [11].

## Results

### SrGAP3 interacts with the CC3 domain of the Slit receptor Robo1 and Robo2 via its SH3 domain

**Figure 1:**
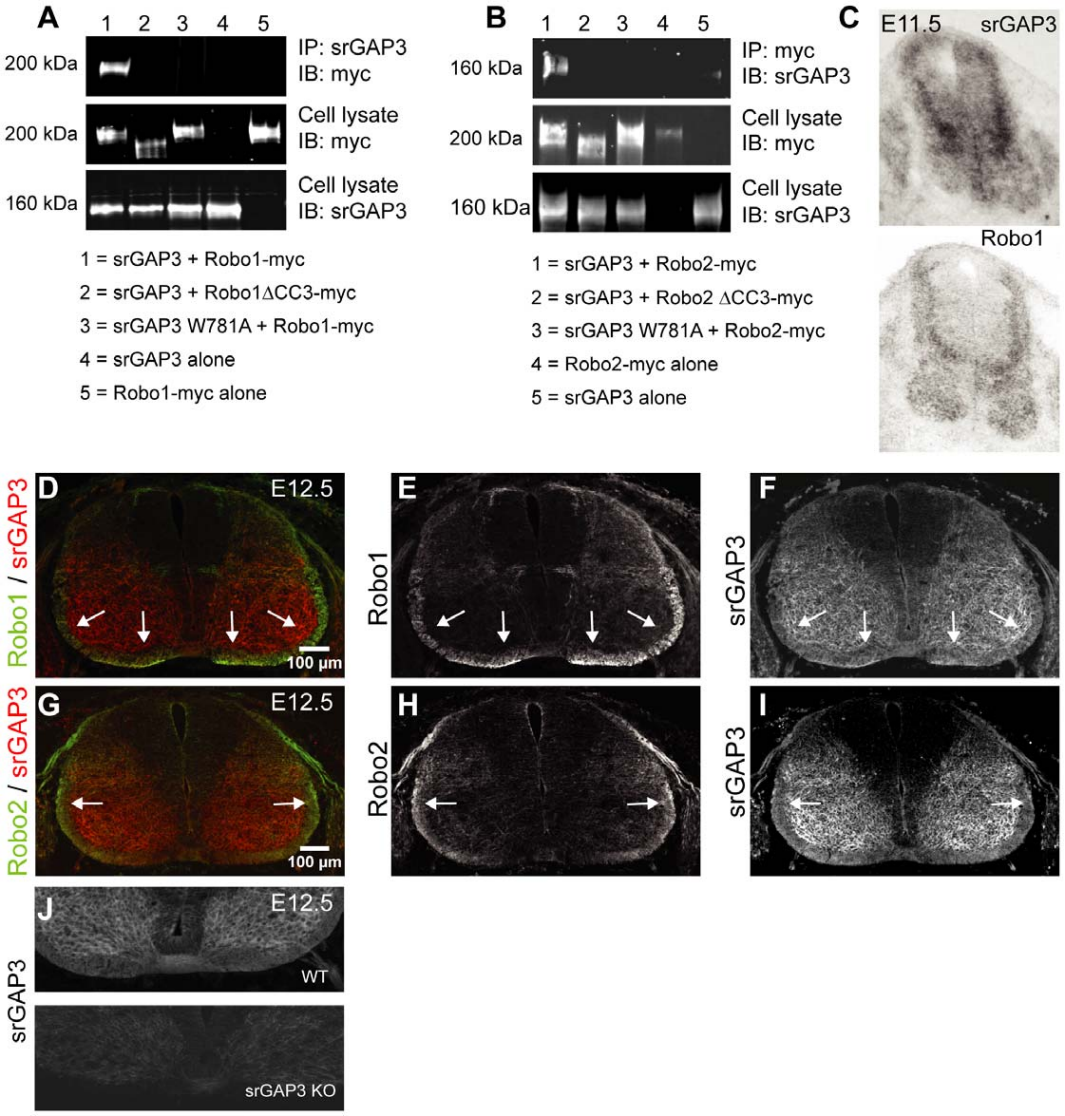
srGAP3 interacts with Robo1 and Robo2 and co-localises with both Robo1 and Robo2 in the spinal cord at E12.5. **A, B:.**HEK293 cells were transfected with srGAP3 and myc-tagged Robo1 or Robo2 contructs and immunoprecipitation revealed that srGAP3 interacts with Robo1 and Robo2 (upper panels, lane 1). No interaction between srGAP3 and Robo1 or Robo2 was observed when HEK293 cells were transfected with a ΔCC3 Robo1 or a ΔCC3 Robo2 construct (upper panels, lane 2) or an srGAP3 construct with a mutation in the SH3 domain (upper panels, lane 3). Control experiments where full length srGAP3, Robo1 or Robo2 were transfected alone into HEK293 cells alone showed no interaction (upper panels, lanes 4 and 5). The middle and lower panels represent the cell lysate immunoblotted with an anti-myc and anti-srGAP3 antibody respectively. **C**: srGAP3 and Robo1 mRNA expression in adjacent transverse sections of E11.5 spinal cords showing that srGAP3 and Robo1 are co-expressed in a subset of neurons in the dorsal spinal cord. **D–F**: srGAP3 and Robo1 immunohistochemistry showing co-localisation of both proteins in the longitudinal axon tracts of the spinal cord at E12.5 (arrows). srGAP3 expression is also detected in the gray matter of the spinal cord (**F, I**). **G–I**: srGAP3 and Robo2 immunohistochemistry showing expression of both proteins in the lateral funiculus of the spinal cord at E12.5 (arrows). **J**: Absence of srGAP3 immunostaining on transverse cryosections of srGAP3 KO spinal cords compared to WT demonstrating specificity of the srGAP3 staining in the spinal cord. Note that the brightness of the panel showing the KO spinal cord was increased in order to see the spinal cord.

Co-immunoprecipitation experiments using HEK293 cells indicated that the SH3 domain of srGAP1 interacts with the CC3 domain of Robo1 [6]. Here, we used the same approach to show that srGAP3 can interact with Robo1 and Robo2 (Figure 1A, B). When we co-transfected a truncated Robo1 or Robo2 construct missing the CC3 domain with full length srGAP3, or full length Robo1 or Robo2 with an srGAP3 construct carrying a mutation in the SH3 domain, we did not observe an interaction (Figure 1A, B). This confirms that the SH3 domain of srGAP3 interacts with the CC3 region of Robo1 and Robo2.

### SrGAP3 protein is localised in longitudinal axon tracts of the spinal cord during commissural axon crossing and co-localises with Robo1 and Robo2

We have shown previously that srGAP3 mRNA is expressed in the spinal cord at E11.5 [11] in a pattern reminiscent to that described for Robo1 [2]. Here we confirm, on adjacent transverse cryosections of E11.5 spinal cord that srGAP3 and Robo1 mRNA are co-expressed in a subset of commissural neurons during midline crossing (Figure 1C). SrGAP3 expression was also observed throughout the entire spinal cord, including ipsilaterally projecting neurons and motor neurons. Next, we showed using immunohistochemistry that srGAP3 is expressed throughout the spinal cord in the gray matter and in the longitudinal axon tracts at E12.5, reflecting the widespread mRNA expression (Figure 1F, I). It is known that Robo1 is expressed throughout the ventrolateral funiculus whereas Robo2 localises exclusively to the lateral funiculus [2]. We were able to confirm this localisation of Robo1 and Robo2 using commercially available antibodies (Figure 1D, E, G, H). Double immunostaining with srGAP3 and Robo1 or Robo2 antibodies showed that srGAP3 and Robo1 are co-expressed in axons of the ventral and lateral funiculus (Figure 1D–F, white arrows) and srGAP3 co-localises with Robo2 in the lateral funiculus (Figure 1G–I, white arrows). SrGAP3 staining was absent in *srGAP3* KO spinal cord sections (Figure 1J), indicating specificity of the staining in the spinal cord.

*In situ* hybridisation for *srGAP1* and *srGAP2* in *srGAP3* KO spinal cords showed that expression of the other srGAP family genes is not altered in the absence of *srGAP3* (Figure S1).

### SrGAP3 is expressed in the majority of TAG-1 positive pre-crossing axons but srGAP3 KO cords have no pre-crossing defects

**Figure 2:**
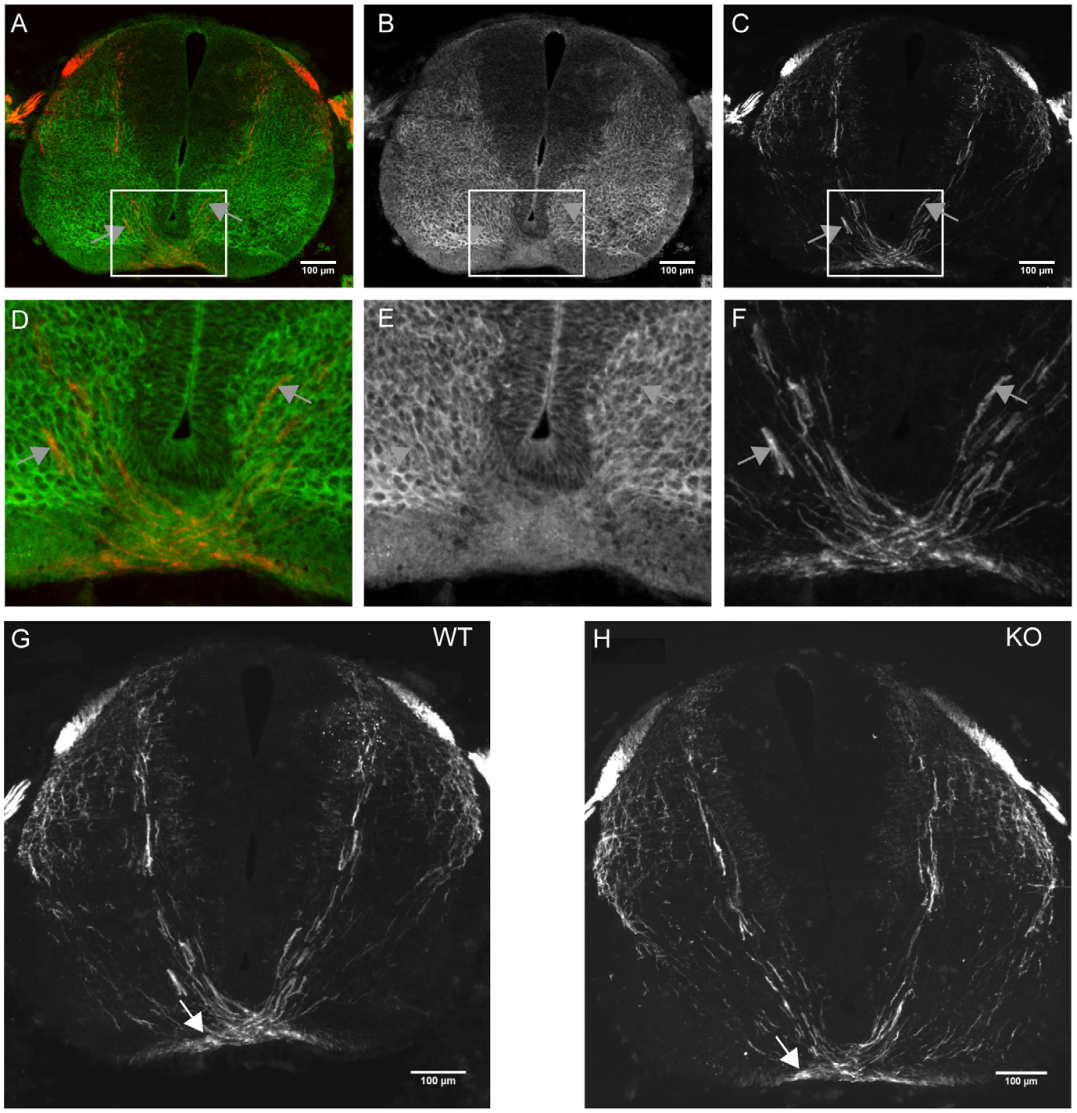
srGAP3 co-localizes with TAG-1 in the majority of commissural axons but TAG-1 positive axons project normally towards the floor plate in srGAP3 KO spinal cords. **A–C**: srGAP3 is expressed in the majority of TAG-1 positive axons (grey arrows). **D–F**: Enlarged images of the areas indicated by white boxes in A–C, showing the floor plate region more clearly and the co-localisation of srGAP3 and TAG-1 (grey arrows) in most axons. **G–H**: TAG-1 immunohistochemistry on WT and srGAP3 KO sections showed that TAG-1 positive axons project normally towards and across the floor plate. TAG-1 staining was also present in the ventral funiculus (white arrows). This was observed in three different srGAP3 KO embryos.

We have shown that srGAP3 protein co-localises with Robo1 in the ventrolateral funiculus and with Robo2 in the lateral funiculus. To examine whether srGAP3 protein also localises to pre-crossing axons we performed double immunohistochemistry of TAG-1 and srGAP3 (Figure 2A–F). SrGAP3 is expressed diffusely throughout the gray matter of the spinal cord and therefore almost all TAG-1 positive axons co-express srGAP3 (Figure 2A–F, grey arrows). In Slit triple mutant mice, TAG-1 positive axons were defasciculated at the floor plate and did not leave the floor plate into the ventral funiculus [2]. However, in the *srGAP3* KO, TAG-1 positive commissural axons projected normally across the floor plate and staining in the ventral funiculus was present (Figure 2G–H, white arrows). This was observed in three different *srGAP3* KO embryos. TAG-1 also labels sensory axons and in Robo1/Robo2 double mutants, TAG-1 positive sensory axons overshoot the dorsal root entry zone [4]. However, we did not observe any overshooting of TAG-1 positive sensory axons in the *srGAP3* KO (Figure 2H).

### Commissural axons do not stall in the floor plate of srGAP3 KO spinal cords

**Figure 3:**
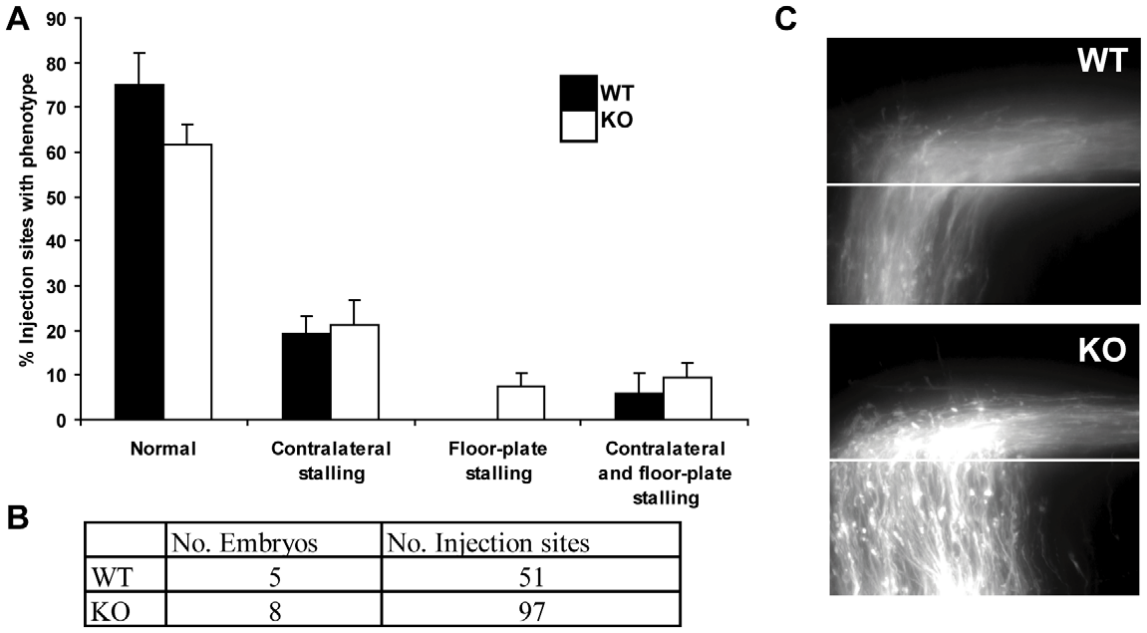
Commissural axons do not stall in srGAP3 KO spinal cords. WT and srGAP3 KO spinal cords were prepared in an open book configuration and dorsolateral commissural axons were traced by the injection of DiI into the dorsal spinal cord. **A**: Injections sites were classified as being either normal, containing >50% of axons stalling in the floor-plate, at the contralateral exit site, or both. No significant differences in the % occurrence of each phenotype were observed between WT and srGAP3 KO cord sections, using the Independent Samples T-test. (P value comparing the % of normal injection sites in WT and srGAP3 KO cords  = 0.12. P value comparing the % of injection sites with floor plate stalling in WT and srGAP3 KO cords  = 0.08). **B**: The number of embryos and the number of injection sites analysed for each genotype is provided in the table. **C**: Representative images of open book preparations injected with DiI showing that axons cross the floor plate normally and project longitudinally after crossing the contralateral exit site (marked by the white line).

Commissural axons stall during crossing in *Robo1* KO, *Robo1/Robo2* double KO and *Slit* triple KO spinal cords [2], [4]. We wanted to determine whether commissural axons also stall in the spinal cord of *srGAP3* KO mice. To do this we injected DiI into the area of the cell bodies of commissural neurons in the dorsal spinal cord of *srGAP3* KO and WT spinal cords. We did not observe axons stalling within the floor plate of KO spinal cords (7% of injection sites, n = 97) significantly more often than in WT spinal cords (0% of injection sites, n = 51, P = 0.077) (Figure 3A). Additionally we found no significant difference in the number of axons stalling at the floor plate exit site in *srGAP3* KO (21%) compared to WT (19%) spinal cords (Figure 3A). Post-crossing trajectories of these subpopulations of commissural neurons did not differ between WT and KO (Figure 3C).

### Loss of srGAP3 affects the size of the ventrolateral funiculus

**Figure 4:**
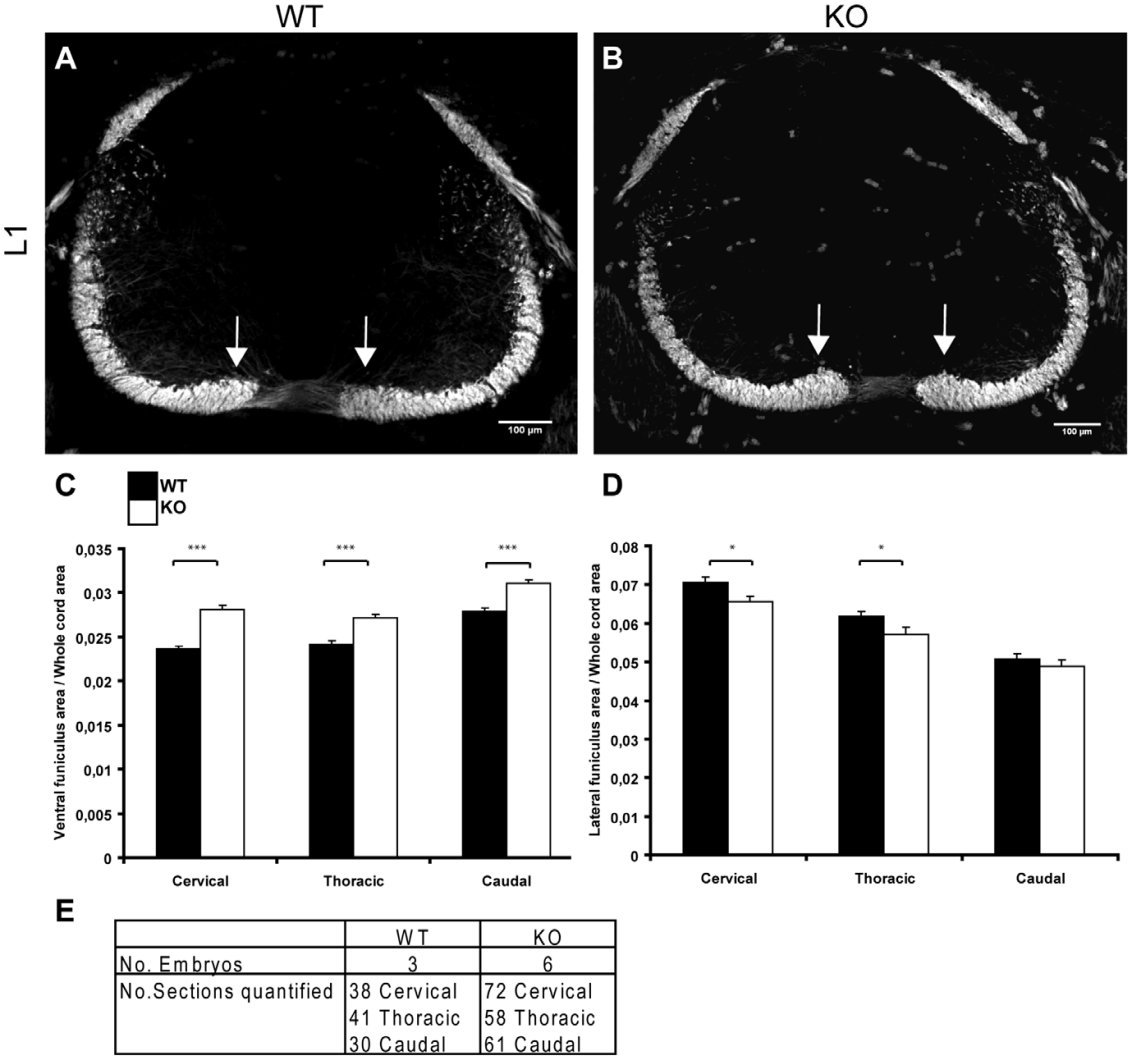
The ventral funiculus of srGAP3 KO spinal cords is significantly thicker compared to WT. **A–B**: L1 staining on transverse sections of srGAP3 WT (**A**) and srGAP3 KO (**B**) spinal cords revealed an enlargement of the ventral funiculus in srGAP3 KO cords compared to WT (white arrows). **C**: The area of the L1-positive ventral funiculus was quantified in WT and srGAP3 KO cord sections at cervical, thoracic and caudal levels and the ventral funiculus of srGAP3 KO spinal cords was found to be significantly thicker at all levels using the Independent Samples T-test (P<0.0001). **D**: The L1 positive lateral funiculus area was also quantified in the same sections and was found to be significantly thinner in srGAP3 KO cervical sections (P = 0.0204) compared to WT and srGAP3 KO thoracic sections (P = 0.0335) compared to WT. The lateral funiculus was also found to be slightly thinner in caudal sections of srGAP3 KO spinal cords compared to WT, but this difference was not significant (P = 0.339). **E**: The number of embryos and sections analysed is provided in the table.

We used L1 staining to visualise the longitudinal axon tracts in E12.5 transverse cryosections (Figure 4A–B). We observed and were able to quantify a thickening of the ventral funiculus next to the floor plate in srGAP3 KO spinal cords, which was absent in WT sections at the same rostro-caudal level (Figure 4A–B, white arrows). We quantified the area of the ventral funiculus (Figure 4C) and the increase in the ventral funiculus was found to be 16% in cervical sections (P  =  <0.0001), 11% in thoracic sections (P  =  <0.0001) and 10% in caudal sections (P  =  <0.0001). Additionally, we quantified the area of the lateral funiculus (Figure 4D) and found a significant thinning of the lateral funiculus in cervical (8% decrease, P = 0.0204) and thoracic (8% decrease, P = 0.0335) sections of *srGAP3* KO spinal cords. The lateral funiculus was also thinner in caudal KO sections compared to caudal WT sections but this was not statistically significant, probably because the lateral funiculus is still in early stages of development at the caudal level (4% thinner, P = 0.339). The total normalised ventrolateral funiculus area was not significantly different between *srGAP3* KO and WT spinal cords (Cervical level: WT (n = 38)  = 0.0942, KO (n = 72)  = 0.0937, P = 0.8; Thoracic level: WT (n = 41)  = 0.0859, KO (n = 58)  = 0.0843, P = 0.52; Caudal level: WT (n = 30)  = 0.0784, KO (n = 61)  = 0.0799, P = 0.6). Additionally, the mean L1 staining intensity did not differ between the KO and the WT spinal cord sections. Quantification of the normalised floor plate commissure thickness (Figure S2) revealed no significant difference between *srGAP3* KO and WT cords (cervical level: WT (n = 22)  = 0.0013, KO (n = 68)  = 0.00142, P = 0.215), indicating that the enlarged ventral funiculus is not the result of more axons crossing the midline.

**Figure 5:**
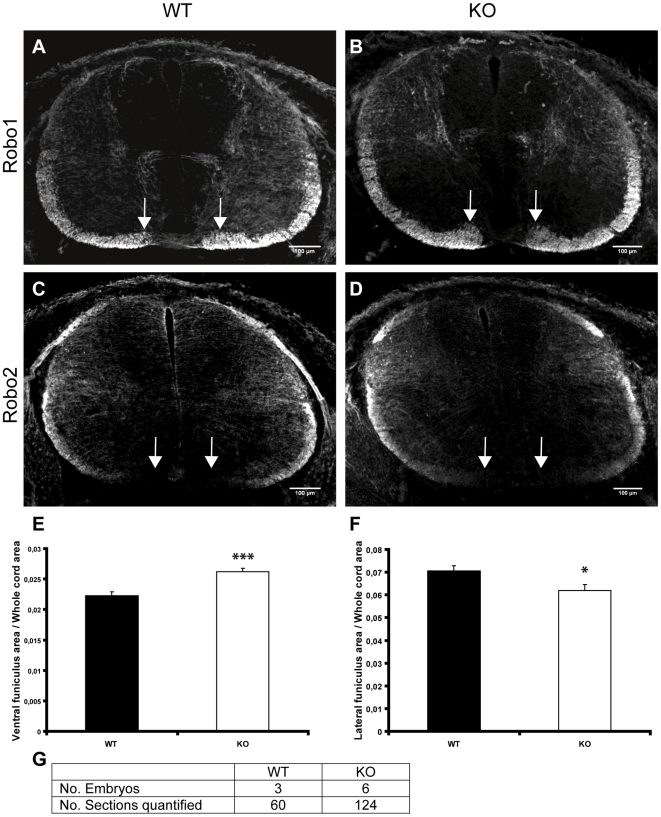
Axons within the enlarged ventral funiculus of the srGAP3 KO are Robo1 but not Robo2 positive. **A**: Robo1 immunohistochemistry on transverse section of E12.5 WT spinal cord, showing the normal size of the Robo1 positive ventral funiculus (white arrows). **B**: The enlarged ventral funiculus in the srGAP3 KO spinal cord is visible with Robo1 immunohistochemistry (white arrows) **C–D**: Immunohistochemistry of Robo2 in WT (**C**) and srGAP3 KO (**D**) cords showing lack of Robo2 expression in the axons of the ventral funiculus in both WT and srGAP3 KO spinal cords (white arrows). **E**: The Robo1 positive ventral funiculus is significantly thicker in srGAP3 KO spinal cords (mean value 0.0262) compared to WT (mean value 0.0223). **F**: The Robo1 positive lateral funiculus was significantly thinner in srGAP3 KO (mean value 0.062) compared to WT (mean value 0.0705) spinal cords (P = 0.0296). Quantifications were performed on cervical sections only. **I**: Summary of the total number of embryos and cervical sections quantified.

The size of the ventrolateral funiculus is altered in *Robo1* and *Robo2* mutants [2], [4]. To investigate the possibility that the thickening of the ventral funiculus in the *srGAP3* KO is a Robo mediated effect, we visualised Robo1 and Robo2 positive axons in WT and srGAP3 KO spinal cords using immunohistochemistry. Robo1 positive axons were found throughout the ventrolateral funiculus in both *srGAP3* KO and WT sections. Interestingly the enlarged ventral funiculus in the *srGAP3* KO was only visible with Robo1 staining (Figure 5A–B; white arrows). No Robo2-positive axons were found in the ventral funiculus (Figure 5C–D; white arrows), as might have been expected based on published results [4]. We quantified the Robo1-positive area of the ventral and lateral funiculi as previously described in cervical spinal cord sections and were able to confirm that the increase of the ventral funiculus and reduction of the lateral funiculus is statistically significant (Figure 5E–F). Additionally, the mean Robo1 staining intensity did not differ between the KO and the WT spinal cord sections.

## Discussion

Commissural axons lose responsiveness to attractive cues secreted by floor plate cells after crossing and instead become receptive to repulsive guidance cues in order to leave the midline. The Slits and their Robo receptors play a key role in repelling axons from the midline. In this paper, we discuss the importance of srGAP3 in the repulsion of commissural axons from the midline, possibly downstream of Slit-Robo.

We have shown that srGAP3 can interact directly with the Slit receptors Robo1 and Robo2, making it a putative signaling factor downstream of Slit. However, loss of srGAP3 does not cause commissural axon stalling within the floor plate as was reported in the Slit triple mutant, the Robo1 mutant and the Robo1/Robo2 double mutant [2], [4]. Most likely, this result can be explained by a redundant role of srGAP2 that is also expressed in commissural neurons of the dorsal spinal cord [11]. Furthermore, there are many other interaction partners of Robo receptors with putative roles in axon repulsion. These include the tyrosine kinase Abelson and its substrate Enabled, both of which can bind directly to Robo and have been shown to have opposite effects on Slit mediated repulsion from the midline in *Drosophila* [12]. Mammalian Ena (Mena) is also required for the normal formation of the corpus callosum and hippocampal commissure in mice [13]. The GEF protein Son of Sevenless and the GAP protein crossGAP/Vilse can both interact with Robo and control Rac activity for midline repulsion downstream of Slit [14], [15]. Additionally, the deubiquinating enzyme USP33 interacts with the Robo1 receptor to regulate Slit-mediated repulsion of commissural axons from the midline in chick [16]. Thus, all these factors could play a role in midline repulsion of commissural axons and explain why the lack of srGAP3 does not cause any crossing defects of commissural axons.

Robo1 and Robo2 localise to distinct portions of the ventrolateral funiculus and appear to control the lateral positioning of longitudinally projecting axons [2], [4], similar to what has been described for Robo2 and Robo3 in Drosophila [17], [18]. Here we show that *srGAP3* KO spinal cords have an enlarged ventral funiculus and a reduced lateral funiculus, suggesting that fewer commissural axons grow laterally away from the floor plate in their longitudinal trajectory. There was no difference in the total area of the ventrolateral funiculus, suggesting that this difference is not simply due to a developmental delay of srGAP3 KO embryos. This has been reported previously in the Robo2 mutant [4] and in the chick spinal cord when a truncated Robo1 construct lacking the cytoplasmic domain was expressed in post crossing commissural axons [19]. However, when we examined the longitudinal axon tracts in DiI injected open book preparations of *srGAP3* KO spinal cords, we did not observe that post-crossing axons projected longitudinally in closer proximity to the floor plate compared to WT cords. Nevertheless, because we injected a very distinct population of commissural neurons in the dorsal spinal cord we cannot exclude the possibility that commissural axons from other neuronal populations would follow a more medial trajectory in the absence of srGAP3.

To address whether the thickening of the ventral funiculus in the *srGAP3* KO spinal cord is a Robo-mediated event, we examined both Robo1 and Robo2 expression in *srGAP3* KO spinal cords. Given that the *srGAP3* KO phenotype is the same as that described for Robo2 [4], we expected to find that this is a Robo2-mediated effect. However, there was no medial shift of Robo2 positive axons into the ventral funiculus in the *srGAP3* KO spinal cord, suggesting that the thickening of the ventral funiculus in the *srGAP3* KO spinal cord is not due to aberrant Robo2 signaling. Instead, we found that the post-crossing axons within the enlarged ventral funiculus are Robo1 positive, which points to the abnormal thickening of the ventral funiculus in the *srGAP3* KO being a Robo1 mediated event. That Robo1 and srGAP3 could be acting in the same pathway to guide commissural axons away from the floor plate is difficult to explain, as the *Robo1* KO has a thinner ventral funiculus, while the *srGAP3* KO has a thicker ventral funiculus. A possible explanation is the occurrence of axon stalling within the floor plate. Commissural axons stall within the floor plate of the *Robo1* KO, which means that fewer axons reach the ventral funiculus [4]. However, we have shown that axon stalling does not occur in the floor-plate of the *srGAP3* KO therefore axons extend into the ventral funiculus. If srGAP3 is involved in Robo1 mediated repulsion from the midline, then our data suggests that it is likely to be after commissural axons have left the floor-plate.

As it has been shown that Robo1 and Robo2 are homophilic adhesion molecules that can interact with each other [20], it is possible that srGAP3 is not required for the Robo-mediated response to Slit but rather as a mediator of Robo/Robo interactions. Therefore, our observation that Robo1-positive axons run more medially in srGAP3 KO spinal cords compared to WT, might not result from a loss of responsiveness to Slit but rather indicate a difference in axonal fasciculation between Robo1 and Robo2-positive axons. In other words, expression of Robo2 might keep axons from extending medially but some axons expressing only Robo1 might not be repelled sufficiently by Slit but also require fasciculation with Robo2-positive axons for their lateral position. Thus, these axons will run more medially in the absence of Robo2 (as shown by Jaworski and colleagues, 2010) or in the absence of srGAP3 (this study).

In conclusion, we have shown that srGAP3 can bind to the Slit receptors Robo1 and Robo2. We have also shown that srGAP3 co-localises with both Robo receptors in the post-crossing longitudinal axons tracts of the spinal cord. Unlike what has been reported in the Slit and Robo mutant mice, we observed no stalling of commissural axons within the floor plate. Instead, we observed a thickening of the ventral funiculus and a reduction of the lateral funiculus, which corresponds to the phenotype described for the *Robo2* KO. However, the axons within the enlarged ventral funiculus were Robo1 but not Robo2 positive. We suggest that srGAP3 is involved in the lateral positioning of commissural axons within the ventrolateral funiculus, possibly downstream of Robo1.

## Supporting Information

Figure S1 mRNA expression of srGAP1 and srGAP2 in the srGAP3 KO spinal cord. To investigate the possibility that srGAP1 or srGAP2 may compensate for the loss of srGAP3 in the srGAP3 KO spinal cord, we analysed the expression of srGAP1 and srGAP2 mRNA in E11.5 srGAP3 KO spinal cords using i*n situ* hybridisation. We found that both srGAP1 and srGAP2 are expressed normally in the srGAP3 KO spinal cord and did not observe any shift in the expression of srGAP1 or srGAP2 into srGAP3 expressing neuronal populations. No staining was observed when *in situ* hybridisation was performed using sense probes for srGAP1 and srGAP2 (data not shown).
(TIF)

Figure S2 The thickness of the floor plate commissure is not altered in srGAP3 KO spinal cords. The floor plate commissure was quantified using ImageJ software. The total length of the L1 positive axon commissure was traced and measured in cervical spinal cord sections. Values were normalised to the total ventrolateral funiculus area. There was no significant difference in the normalised floor plate thickness between srGAP3 KO and WT spinal cords.
(TIF)
