# Supplementary figures and images for: Evidence for a Role of srGAP3 in the Positioning of Commissural Axons within the Ventrolateral Funiculus of the Mouse Spinal Cord
Source: PLoS One. 2011 May 31;6(5):e19887. doi: 10.1371/journal.pone.0019887 (PMC3104994; doi:10.1371/journal.pone.0019887)

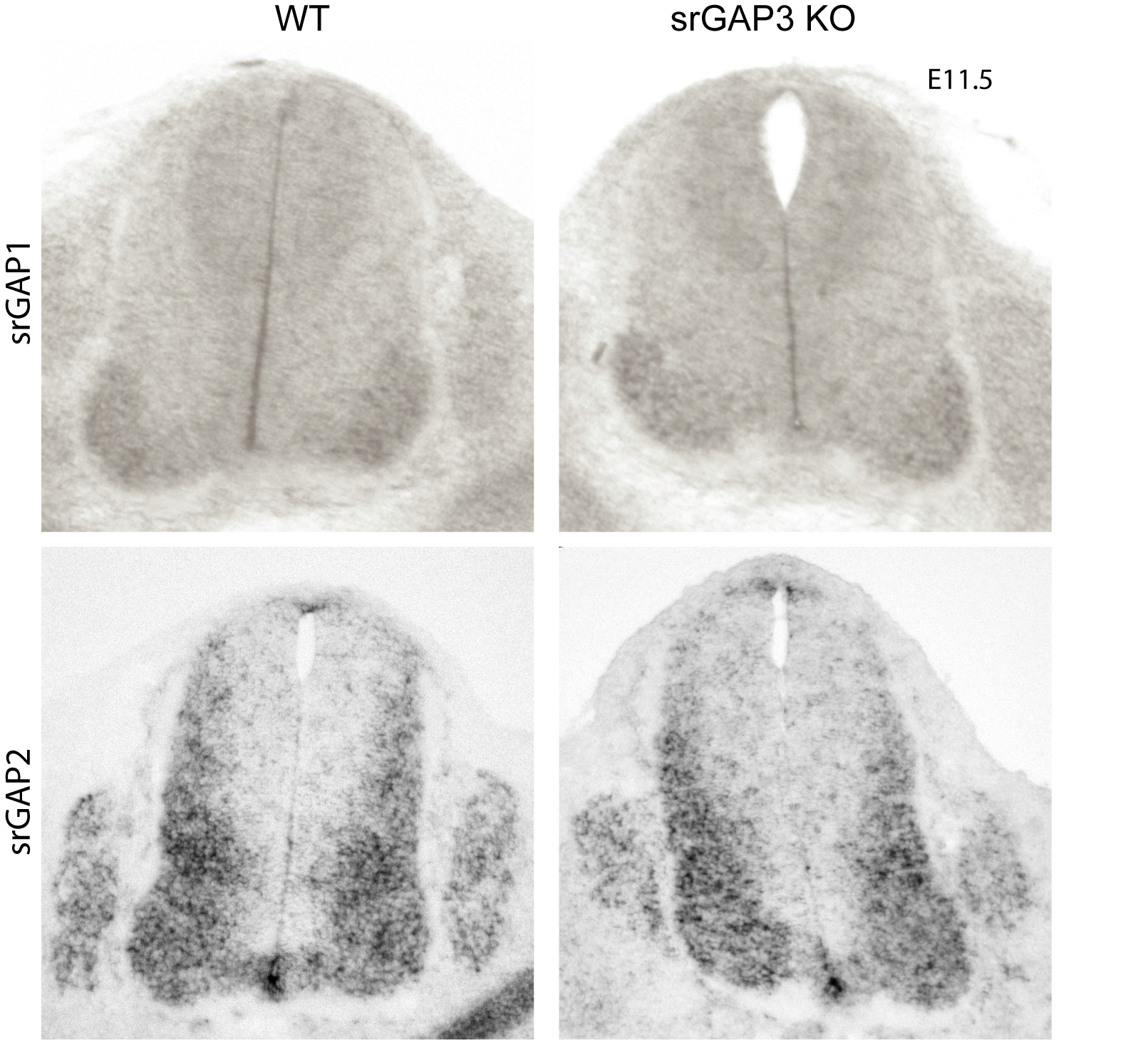

Supplement: Figure S1 — mRNA expression of srGAP1 and srGAP2 in the srGAP3 KO spinal cord. To investigate the possibility that srGAP1 or srGAP2 may compensate for the loss of srGAP3 in the srGAP3 KO spinal cord, we analysed the expression of srGAP1 and srGAP2 mRNA in E11.5 srGAP3 KO spinal cords using in situ hybridisation. We found that both srGAP1 and srGAP2 are expressed normally in the srGAP3 KO spinal cord and did not observe any shift in the expression of srGAP1 or srGAP2 into srGAP3 expressing neuronal populations. No staining was observed when in situ hybridisation was performed using sense probes for srGAP1 and srGAP2 (data not shown). (TIF) [file pone.0019887.s001.tif]

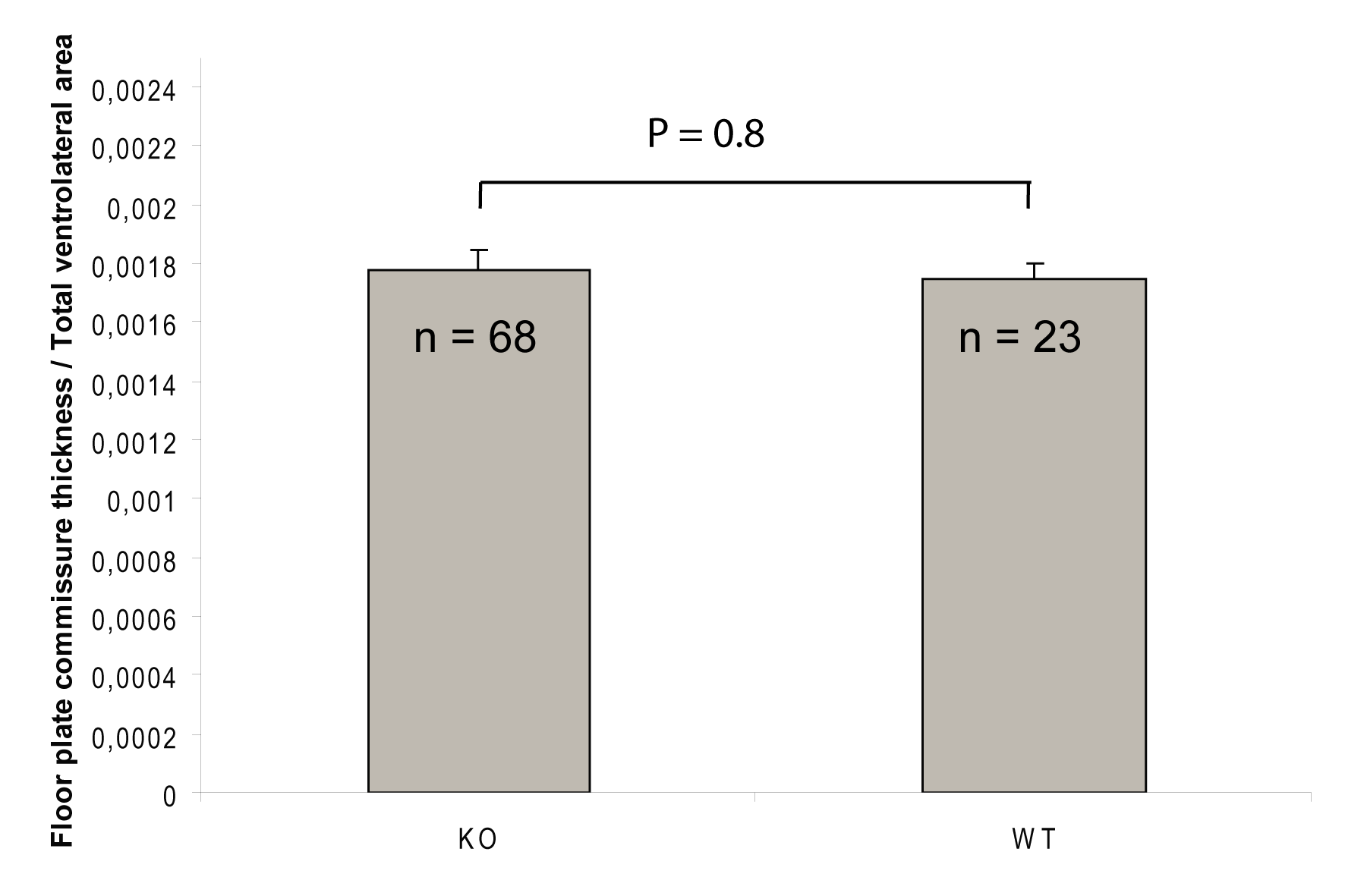

Supplement: Figure S2 — The thickness of the floor plate commissure is not altered in srGAP3 KO spinal cords. The floor plate commissure was quantified using ImageJ software. The total length of the L1 positive axon commissure was traced and measured in cervical spinal cord sections. Values were normalised to the total ventrolateral funiculus area. There was no significant difference in the normalised floor plate thickness between srGAP3 KO and WT spinal cords. (TIF) [file pone.0019887.s002.tif]
